# The Impact of Non-opioid Analgesic Usage on Total Opioid Load During Traumatic Brain Injury Rehabilitation: A Retrospective Study

**DOI:** 10.7759/cureus.46872

**Published:** 2023-10-11

**Authors:** Joshua D King, Kylo Cautivar, Duc A Tran, Mohammad Ali, Nicholas Schmidt, Michelle McSkane, Eugene Pak

**Affiliations:** 1 Physical Medicine and Rehabilitation, Loma Linda University Medical Center, Loma Linda, USA; 2 Pain Medicine, Loma Linda University Medical Center, Loma Linda, USA

**Keywords:** methocarbamol, lidocaine patch, ibuprofen, gabapentin, diclofenac gel, baclofen, aspirin, amitriptyline, acute inpatient rehabilitation, acetaminophen

## Abstract

Background

Patients staying in acute rehabilitation often use large amounts of opioids during their stay. There are a number of reasons for this increased opioid exposure, including but not limited to daily exercises with physical and occupational therapists, increased demand on a healing body, and use of previously atrophying musculature. Some physiatrists have noticed that patients who concurrently are prescribed medications such as Robaxin seem to require fewer opioids during their stay in acute rehabilitation. This study aimed to determine the association between non-opioid analgesic use and total opioid load, as measured using morphine milligram equivalents (MMEs), during inpatient rehabilitation for traumatic brain injury.

Methodology

A retrospective study of individuals with a diagnosis of traumatic brain injury admitted to an acute inpatient rehabilitation program was performed. Non-opioid medications that were reviewed in the study included acetaminophen, amitriptyline, baclofen, diclofenac, gabapentin, ibuprofen, lidocaine, methocarbamol, nortriptyline, and pregabalin. Five of the most-used non-opioid medications (acetaminophen, diclofenac, gabapentin, lidocaine, and methocarbamol) were statistically analyzed using regression and analysis of variance to evaluate for any significant variables.

Results

Results showed that the average daily dose of acetaminophen has a significant effect on the average daily MME and that the average daily dose of gabapentin and methocarbamol each have a significant effect on the change of daily MME usage from admission to discharge from acute rehab (ΔMME). Results also showed that the mere presence of methocarbamol (regardless of daily or total dosage) had a significant effect on the ΔMME.

Conclusions

Based on these findings, physicians may want to consider prescribing acetaminophen, gabapentin, or methocarbamol for patients admitted for inpatient rehabilitation following traumatic brain injury who require high amounts of opioids.

## Introduction

The opioid crisis in the United States began in the 1990s after OxyContin was introduced and advertised as a safe and effective analgesic. At that time, Purdue Pharma, a pharmaceutical company founded and owned by the Sackler family, was pursuing a vigorous campaign to promote the more liberal use of opioids for pain [1]. Initiatives like the Joint Commission began to push for more attentive physician responses to patient pain, referring to pain as the fifth vital sign. This exacerbated the already increasing number of opioids being prescribed by doctors to patients [2]. Between 1991 and 2011, prescriptions for painkillers in the United States increased from 76 million to 219 million per year. In 2016, 289 million prescriptions were written for opioids [3]. From 1999 to 2016, it is estimated that 453,300 Americans have died from opioid use [4].

This crisis necessitates a response from physicians. With respect to the field of physiatry, physiatrists are in a unique position. Many patients admitted to in-patient rehabilitation arrive days after undergoing a large surgery, involvement in a motor vehicle accident, or injury from a life-changing medical event such as a stroke or brain injury.

Inpatient rehabilitation of patients following traumatic brain injuries (TBIs) often involves some level of opioid load, and as such this population is at risk for opioid overuse and addiction. It is important to recognize that TBI patients often present with more than just brain injuries when they arrive at the hospital. In fact, a significant proportion of these patients also experience concurrent extremity injuries. Data have shown that approximately 40% to 60% of TBI patients exhibit coexisting injuries to their extremities and many require surgical intervention [5-7]. Patients with traumatic injuries are at a higher risk for chronic opioid use [8]. Studies indicate that more than 50% of individuals living with TBI have chronic pain [9,10]. TBI patients are at a greater risk of developing substance abuse as well as having difficulty in substance abuse programs given their decreased executive function [11-13]. Considering the potential risk, it is prudent for physiatrists to consider alternative methods of managing pain, as well as to investigate if specific combinations of medications are able to reduce overall opioid exposure while still providing enough pain reduction to allow patient participation in therapies.

It has been hypothesized by some physiatrists that patients who are concurrently prescribed common non-opioid analgesics (such as gabapentin) with their opioids seem to require fewer opioids during their inpatient rehabilitation admissions. The incidence and prevalence of TBI population continue to grow globally and domestically [14,15]. It is now important more than ever to look at alternatives to opioid use in inpatient rehabilitation settings. This study aimed to determine the association between non-opioid analgesic use and total opioid load (measured using morphine milligram equivalents (MMEs)) during inpatient rehabilitation for brain injury patients.

## Materials and methods

Study design and data source

The study was designed as a retrospective chart review. The study design was submitted to Loma Linda University’s institutional review board (IRB), Human Research and Compliance. The study design was given IRB# 5190504 and was approved by the board. After IRB approval was obtained, the intake department manager for inpatient rehabilitation and the coordinator of rehabilitation management were contacted and provided access to an on-site protected database with a list of all admissions to inpatient rehabilitation. This database was queried for patients who fit the following criteria: (1) were admitted from January 1, 2019, to December 31, 2019; (2) were admitted with “brain injury” as the rehabilitation diagnosis; and (3) were safely discharged from the service (did not expire during in-patient stay). In total, 42 patients met these criteria and were included in the final study. Each of these patients’ charts was accessed safely from on-site protected computers, and a de-identified database was constructed.

Variables

The following information was obtained and recorded for each patient from his or her chart: gender, age, length of stay in days, total MMEs, daily MMEs on admission, daily MMEs on discharge, and average daily MMEs. With respect to non-opioid analgesics, the following data was recorded (in milligrams): total dose, daily dose on admission, daily dose on discharge, and average daily dose. The presence of the following non-opioid analgesics during rehabilitation admission was queried for each patient: acetaminophen, amitriptyline, aspirin, baclofen, capsaicin, celecoxib, cyclobenzaprine, desvenlafaxine, diclofenac gel, duloxetine, gabapentin, ibuprofen, ketorolac, lidocaine patch, meloxicam, methocarbamol, milnacipran, naproxen, nortriptyline, pregabalin, tizanidine, and venlafaxine. Of these, the following were found to be present: acetaminophen, amitriptyline, aspirin, baclofen, diclofenac gel, gabapentin, ibuprofen, lidocaine patch, methocarbamol, nortriptyline.

Statistical analysis

**Figure 1:**
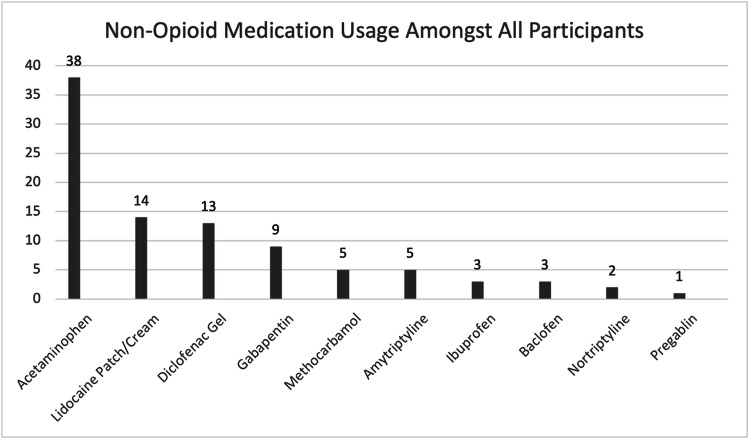
Number of patients using each non-opioid analgesic during admission. Acetaminophen, lidocaine patch, diclofenac gel, gabapentin, and methocarbamol were selected for analysis using regression and analysis of variance.

After the data was collected, it was found that the following non-opioid analgesics were given to most patients: acetaminophen, lidocaine patch, diclofenac gel, gabapentin, and methocarbamol (Figure 1). Statistical analysis of these five medications using regression and analysis of variance (ANOVA) was performed using a statistical program and with the assistance of the research director for the Physical Medicine and Rehabilitation department at Loma Linda University Hospital. The results of these analyses were then exported from the program and converted into a readable format.

## Results

Patient characteristics

**Figure 2:**
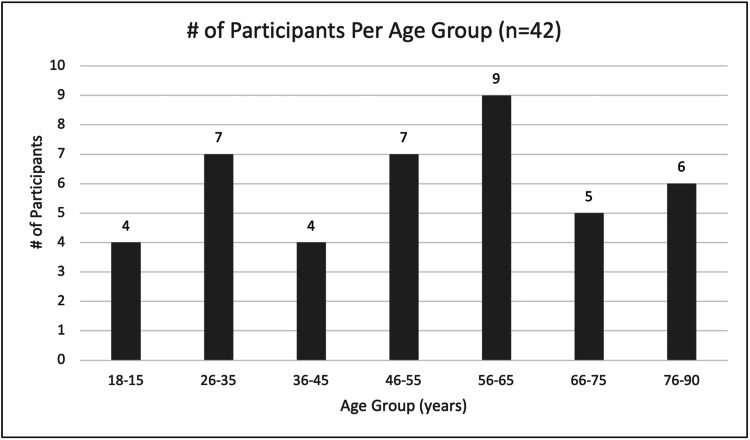
Age of patients on admission. The average age of 52.2 years. Youngest patient: 18 years old. Oldest patient: 89 years old.

**Figure 3:**
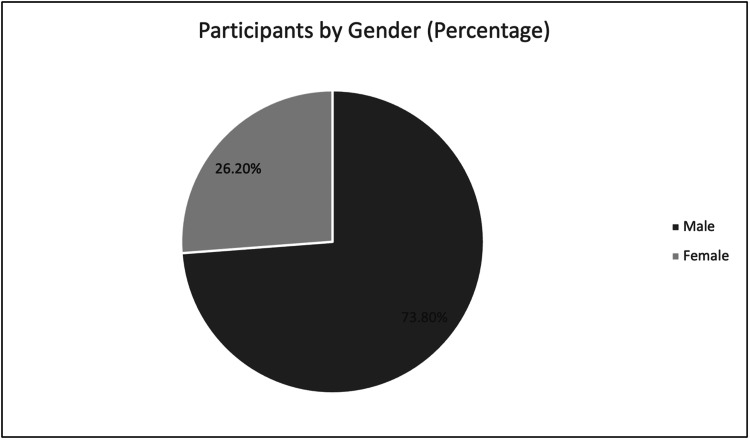
Gender of patients. Males: 31. Females: 11.

With respect to the patient population, patients ranged in age from 18 to 89 years old, with an average age of approximately 52 years (Figure 2). With respect to gender, the population skewed male, with 31 male patients and only 11 female patients (Figure 3). Length of stay ranged from 1 to 48 days, with an average stay of approximately 16 days.

Non-opioid analgesic usage significance

Statistical analysis using regression revealed that the average daily dose (measured in milligrams) of acetaminophen had a significant effect on the average daily MME (Table 1) and that the average daily dose of gabapentin and methocarbamol had a significant effect on the normalized reduction in MMEs throughout the admission (Table 2). Statistical analysis using ANOVA revealed that the presence of methocarbamol had a significant effect on the normalized reduction in MMEs throughout the admission (Table 3).

**Table 1:** Linear regression analysis of dose of non-opioid analgesic on average daily MMEs. SE = standard error; t = t stats; P = P-value; Cl = confidence interval; MME = morphine milligram equivalent

	SE	t	P > t	95% CI	95% CI
Average daily dose of gabapentin (571.0 mg)	0.0118047	0.23	0.82	-0.0212284	0.0266537
Average daily dose of acetaminophen (761.5 mg)	0.0063674	2.38	0.023	0.0022615	0.028089
Average daily dose of diclofenac (4.5 g)	1.912477	0.04	0.972	-3.810373	3.946992
Average daily dose of lidocaine (1.0 patches)	8.945282	-1.39	0.174	-30.54888	5.734867
Average daily dose of methocarbamol (1,781.5 mg)	0.0084291	1.59	0.12	-0.0036575	0.0305324

**Table 2:** Linear regression analysis of dose of non-opioid analgesic on MME reduction from admission to discharge. SE = standard error; t = t stats; P = P-value; Cl = confidence interval; MME = morphine milligram equivalent

	SE	t	P > t	95% CI	95% CI
Average daily dose of gabapentin (571.0 mg)	0.0003033	3.45	0.001	0.0004312	0.0016615
Average daily dose of acetaminophen (761.5 mg)	0.0001636	-0.13	0.896	-0.0003533	0.0003103
Average daily dose of diclofenac (4.5 g)	0.0491382	0.6	0.552	-0.0701222	0.1291915
Average daily dose of lidocaine (1.0 patches)	0.2298355	-0.82	0.419	-0.6541271	0.2781289
Average daily dose of methocarbamol (1,781.5 mg)	0.0002166	4.68	0	0.0005738	0.0014522

**Table 3:** ANOVA of the presence of non-opioid analgesic on MME delta from admission to discharge. SS = sum of squares; dF = degrees of freedom; MS = mean squares; F = F ratio; Prob = probability; MME = morphine milligram equivalent; ANOVA = analysis of variance

	Partial SS	dF	MS	F	Prob > F
Model	21.187503	5	4.2375006	8.19	0
Presence of acetaminophen	0.31476176	1	0.314761764	0.61	0.4406
Presence of diclofenac	1.5316409	1	1.5316409	2.96	0.094
Presence of gabapentin	0.93143916	1	0.931439157	1.8	0.1882
Presence of lidocaine	2.185326	1	2.185326	4.22	0.0472
Presence of methocarbamol	18.9377392	1	18.9377392	36.59	0
Residual	18.6335703	36	0.517599176		
Total	39.8210733	41	0.97124569		

## Discussion

The findings of this study highlight the potential of non-opioid analgesic interventions, namely, acetaminophen, gabapentin, and methocarbamol, to significantly reduce the overall opioid load for TBI patients during acute inpatient rehabilitation. The statistically significant results suggest that these specific interventions have the potential to serve as effective alternatives to opioids in managing pain among TBI patients. By reducing the reliance on opioids, these non-opioid interventions may help mitigate the risk of opioid-related adverse effects and contribute to improved patient outcomes.

Examining the long-term mortality, morbidity, and cost-effectiveness of non-opioid interventions compared to opioid interventions in brain injury patients would provide valuable insights into the overall impact of these treatment approaches. Understanding the extended outcomes associated with these interventions is crucial for optimizing patient care, informing clinical decision-making, and allocating healthcare resources effectively.

First, investigating the long-term mortality rates associated with non-opioid interventions versus opioid interventions is essential. Brain injury patients are already at an increased risk of mortality, and the use of opioids for pain management may further compound this risk due to potential adverse effects, such as respiratory depression and sedation. By evaluating mortality rates over an extended period, researchers can assess whether non-opioid interventions offer any advantages in terms of reducing mortality or minimizing the potential risks associated with opioids. A comprehensive understanding of the long-term mortality outcomes would help guide clinicians in selecting the most appropriate analgesic interventions for brain injury patients, prioritizing patient safety, and optimizing survival rates.

In addition to mortality, investigating long-term morbidity outcomes is crucial. Brain injury patients often face complex and multifaceted challenges, including cognitive impairments, functional limitations, and psychological difficulties. These factors significantly impact their quality of life and overall well-being. By comparing the long-term morbidity outcomes between non-opioid and opioid interventions, researchers can assess whether one approach offers better pain management and contributes to improved functional recovery, cognitive outcomes, and psychological well-being. Such insights would aid healthcare providers in tailoring treatment plans that prioritize comprehensive rehabilitation and address the long-term needs of brain injury patients, ultimately enhancing their overall functional outcomes and quality of life.

Furthermore, considering the cost-effectiveness of non-opioid interventions in comparison to opioid interventions is of utmost importance. Healthcare systems globally are under increasing pressure to allocate resources efficiently. Evaluating the cost-effectiveness of these interventions over the long term would provide valuable information for healthcare policymakers and payers. By comprehensively assessing the economic impact of non-opioid interventions, decision-makers can make informed choices regarding resource allocation, ensuring that healthcare resources are directed toward interventions that deliver the greatest value in terms of both clinical outcomes and cost efficiency.

However, it is crucial to acknowledge the limitations of the current study, particularly its small sample size of only 42 patients. The small sample size may limit the generalizability of the results and increase the likelihood of chance findings. Future studies with larger sample sizes are warranted to provide a more robust assessment of the efficacy and safety of non-opioid analgesics in the TBI population. By increasing the sample size, researchers can enhance the statistical power of the study, enabling them to draw more definitive conclusions and increase the reliability of the findings.

Moreover, it is important to address the issue of medications that were not taken by any patients in the study. The absence of patients taking these medications limits the ability to draw conclusions about their efficacy in reducing opioid load for TBI patients. To gain a more comprehensive understanding of non-opioid interventions, future studies should include a broader range of medications and investigate their impact on pain management outcomes. By incorporating a larger variety of non-opioid analgesics, researchers can explore the potential benefits of different medications and identify the most effective options for pain relief in TBI patients.

Furthermore, it is worth noting the heterogeneity of the patient population in terms of the severity of trauma and the number of injuries sustained. While a rehabilitation diagnosis of “brain injury” was required for each patient, the variability in injury severity and the number of injuries introduces a level of complexity to the study results. The heterogeneous nature of the patient population may influence the response to non-opioid interventions and potentially affect the overall outcomes observed. To address this, future research endeavors should consider stratifying patients based on injury severity and other relevant factors. This approach will enable a more nuanced analysis of the effects of non-opioid analgesics on different subgroups within the TBI population and provide valuable insights into the optimal pain management strategies for each subgroup.

## Conclusions

Based on these findings, physicians may want to consider prescribing acetaminophen, gabapentin, or methocarbamol for patients admitted for inpatient rehabilitation following TBI who require high amounts of opioids. More research is needed, preferably with larger sample sizes, to provide greater clarity. The goal of optimizing patient care to reduce opioid load while maintaining the highest quality of recovery possible is one that should continue to be sought after.

## References

[1] Van Zee A (2009). The promotion and marketing of oxycontin: commercial triumph, public health tragedy. Am J Public Health.

[2] Baker DW (2017). History of the Joint Commission's pain standards: lessons for today's prescription opioid epidemic. JAMA.

[3] (2023). Addiction and substance misuse reports and publications. U.S. Surgeon General.

[4] (2023). The opioid epidemic might be much worse than we thought. https://www.theatlantic.com/health/archive/2020/02/more-people-have-died-opioids-us-thought/607165/.

[5] Kushwaha VP, Garland DG (1998). Extremity fractures in the patient with a traumatic brain injury. J Am Acad Orthop Surg.

[6] Garland DE, Rhoades ME (1978). Orthopedic management of brain-injured adults. Part II. Clin Orthop Relat Res.

[7] Groswasser Z, Cohen M, Blankstein E (1990). Polytrauma associated with traumatic brain injury: incidence, nature and impact on rehabilitation outcome. Brain Inj.

[8] Mauck MC, Zhao Y, Goetzinger AM (2023). Incidence of persistent opioid use following traumatic injury [in press]. Reg Anesth Pain Med.

[9] Nampiaparampil DE (2008). Prevalence of chronic pain after traumatic brain injury: a systematic review. JAMA.

[10] Cifu DX, Taylor BC, Carne WF, Bidelspach D, Sayer NA, Scholten J, Campbell EH (2013). Traumatic brain injury, posttraumatic stress disorder, and pain diagnoses in OIF/OEF/OND Veterans. J Rehabil Res Dev.

[11] Adams RS, Corrigan JD, Dams-O'Connor K (2020). Opioid use among individuals with traumatic brain injury: a perfect storm?. J Neurotrauma.

[12] Corrigan JD, Cole TB (2008). Substance use disorders and clinical management of traumatic brain injury and posttraumatic stress disorder. JAMA.

[13] Corrigan JD, Adams RS (2019). The intersection of lifetime history of traumatic brain injury and the opioid epidemic. Addict Behav.

[14] (2017). Global, regional, and national incidence, prevalence, and years lived with disability for 328 diseases and injuries for 195 countries, 1990-2016: a systematic analysis for the Global Burden of Disease Study 2016. Lancet.

[15] Corrigan JD (2015). TBI at the Centers for Disease Control and Prevention. J Head Trauma Rehabil.

